# Affordability of Canada's Food Guide: Current challenges amid COVID-19, War in Ukraine, and other world events

**DOI:** 10.3389/fnut.2023.1085855

**Published:** 2023-03-30

**Authors:** Stacey Taylor, Sylvain Charlebois, Janet Music

**Affiliations:** ^1^Faculty of Computer Science, Dalhousie University, Halifax, NS, Canada; ^2^Agri-Food Analytics Lab, Dalhousie University, Halifax, NS, Canada; ^3^Faculty of Arts and Social Sciences, Dalhousie University, Halifax, NS, Canada

**Keywords:** Canada's Food Guide, cost analysis, food insecurity, policy for food, COVID-19

## Abstract

Since the 2019 Canada Food Guide was released, there have been concerns raised over the cost of food, with an emphasis on the affordability of nutritious food. In this study, we evaluate the affordability of the 2019 Canada Food Guide in relation to the previous edition from 2007. As a result of the pandemic and other significant world events, many are feeling financial stress as prices in many areas of life rise, including housing, gas, and food. Our results show that it is more cost-effective, on average, for children and teens to follow the 2019 Canada Food Guide, but more expensive for adults, when compared to the 2007 edition.

## 1. Introduction

The first edition of the Canada Food Guide (CFG) was released in 1942 and provided information on food that both adults and children should be consuming each day as well as recommended portion size as part of a nutritious diet (1). Since then, every food guide has provided this information to users. However, in 2019, there was a sharp departure from this approach, instead promoting *mindful eating* by using a plate analogy where each meal should comprise 50% fruits and vegetables, 25% grain, and 25% protein (2). Unlike previous guides, the current guide does not provide recommended portion size or how much food people should be eating per day.

While this approach may encourage better food habits than previous guides, it creates several unresolved challenges. Without suggested portion sizes, users are left to use their own judgment, which creates subjectivity for quantities and proportions; a plate may still resemble the portions outlined in the CFG, but how much is 25% grain, for example? A loaded or scant helping can still fit on 25% of the plate but could be significantly larger or smaller than diet experts would encourage.

Another significant issue is that the CFG is not only used to make good choices on a personal or familial level; it is also used on an institutional level, where hospitals, care homes, corrections facilities, and schools, for example, use these guidelines to purchase food for their operations (3). Without suggested portion sizes and daily food intake, it is difficult for individuals and institutions to cost out healthy diets and create reasonable budgets; the practicality of the guide itself is challenging.

While the CFG's new holistic approach may be helpful from a visual and health perspective, it is very problematic from a costing and budgeting perspective. There is also no evidence that Health Canada considered cost in any meaningful way (4) when developing the guidelines, implying to CFG users that cost should not be a factor.^1^ *That should not and cannot be the case*. There are many stakeholders that may use the CFG to guide their food purchasing choices ranging from individuals to families to institutions (such as nursing homes and hospitals), where cost is a major factor. The ability to forecast and look for alternatives is very hampered without recommended portion sizes.

The CFG is a prominent and well-known document. It is the second most downloaded document provided by the Canadian government which strongly supports that there is public awareness of the guide and has a potential influencing factor on food choice (4). Shortly after the release of the 2019 CFG, Charlebois *et al*. conducted a study on the affordability of the new food guide and found that it was more cost-effective than the previous version (4). However, the affordability of food during the pandemic and in the recent post-pandemic era has raised questions as to whether the findings of Charlebois et al. still hold. The purpose of this study is to evaluate if the 2019 CFG remains more cost-effective than the 2007 version, given recent world events.

The rest of our article is organized as follows: Section 2 provides method and data; Section 3 discusses the results; Section 4 provides a discussion that orients our results among the broader literature; and Section 5 wraps up the research with the conclusion and future work.

## 2. Method and data

The average monthly retail prices for food and other select products was gathered from Statistics Canada^2^ from January 2018 to February 2022.^3^ Canada (5) the foods that Statistics Canada tracks are staples. Proteins include different cuts of meat (beef, chicken, and pork), canned salmon, milk products, cheese slices, peanut butter, baked beans, and eggs. Grains include soda crackers, flour, bread, macaroni, and cornflakes. Only three fruits are tracked by Statistics Canada: apples, bananas, and oranges. Vegetables include carrots, mushrooms, onions, potatoes, tomatoes, and French fries. Using the list, foods were then allocated to the three categories on the CFG plate—fruits and vegetables, proteins, and grains. Food items listed such as soup, baby food, sugar, and ketchup, for example, which did not correspond to any of the three categories, were removed from the dataset. Juices were also removed as the 2019 CFG encourages the consumption of “whole or cut vegetables and fruits instead of juice” (2).

Statistics Canada's data are tracked in various measurements depending on the food: kilograms, grams, liters, milliliters, and by the dozen for eggs. The 2007 CFG uses grams, milliliters, cups, ounces, tablespoons, and individual eggs. To ensure the consistency of portion sizes vs. the costs tracked by Statistics Canada, only grams, milliliters, and individual eggs were used for costing purposes. Costs were converted to per gram, per milliliter, and per individual egg basis. For the 2007 CFG, protein and milk were assessed as two different categories, and the average cost of each item was calculated separately. For the 2019 CFG, milk and proteins were added together before calculating the average cost. This method was used because the approaches between the 2007 and 2019 CFGs changed in that two food groups were merged: *Milk and Alternatives* amalgamated with *Meat and Alternatives* to form *Proteins for the 2019 CFG*. The average cost for each category (protein, milk, grain, and fruits/vegetables) was then calculated and multiplied by the suggested daily servings to determine the cost per day for each category.

The 2007 CFG had four food groups consisting of fruits and vegetables, grain products, milk and alternatives, and meat and alternatives. The 2019 guide only has three food groups—fruits and vegetables, grains, and protein. As the 2019 CFG does not provide serving sizes [as has been provided in all previous CFGs from 1942 to 2007 (1)], the number of overall servings for each group was preserved and then re-allocated from four food groups to three.

Table 1 shows the conversion from the 2007 CFG to the 2019 CFG, broken down by age and gender, as well as food category (fruits and vegetables, grains, milk and alternatives, and meat and alternatives). Table 1 also shows that there is no change in the daily total of food portions and that the amounts from 2007 have been re-apportioned in line with the 2019 requirements of 50% fruits and vegetables, 25% grain, and 25% protein. To perform this conversion, we assumed that the intention of the 2019 CFG was not to reduce the overall amount of food consumed per day but rather to apportion it differently between the three food groups.

**Table 1:** This table shows the conversation from the 2007 CFG to the 2019 CFG, broken down by age and gender, as well as food category (fruits and vegetables, grain milk and alternatives, and meat and alternatives).

	**Conversation of portions from 2007 to 2019**							
	**2–3 yrs**	**4–13 yrs**	**Teen (F)**	**Teen (M)**	**Adult (F) 19–50**	**Adult (M) 19–50**	**Adult (F) 50**+	**Adult (M) 50**+
**2007**				
Fruit, Veg	4	6	8	10	8	10	8	10
Grain	3	6	7	8	7	8	7	8
Milk, Alt	2	4	4	4	2	2	3	3
Meat, Alt	1	2	2	3	2	3	2	3
Total	10	18	21	25	19	23	20	24
**2019**				
Fruit, Veg	5	9	10.5	12.5	9.5	11.5	10	12
Grain	2.5	4.5	5.25	6.25	4.75	5.75	5	6
Protein	2.5	4.5	5.25	6.25	4.75	5.75	5	6
Total	10	18	21	25	19	23	20	24

It also shows that there is no change in the daily total of food portions, and that the amounts from 2007 have been re-apportioned in line with the 2019 requirements of 50% fruits and vegetables, 25% grain, and 25% protein.

## 3. Results

As shown in Tables 2, 3, our findings indicate that it is more affordable for children and teens to follow the 2019 food guide when portions are converted using the 2007 guide as a benchmark. The highest cost among all age categories is male teenagers who, by portion, consume the most—a total of 25 portions per day. Therefore, it would make sense for this age category to have the highest cost. Under the 2007 guide, the cost for a male teenager per day was $9.12, whereas it was only $8.78 using the 2019 guide.

**Table 2:** This table provides the average daily cost (in dollars) for each group, as defined in the 2007 Canada Food Guide.

	**Average daily cost using the 2007 Canada food guide in $**							
	**2–3 yrs**	**4–13 yrs**	**Teen (F)**	**Teen (M)**	**Adult (F) 19–50**	**Adult (M) 19–50**	**Adult (F) 50**+	**Adult (M) 50**+
2018	3.47	6.48	7.09	8.32	**5.62**	**6.84**	**6.35**	**7.58**
2019	3.55	6.63	7.27	8.54	**5.78**	**7.05**	**6.52**	**7.79**
2020	3.67	6.86	7.50	8.82	**5.97**	**7.29**	**6.74**	**8.06**
2021	3.79	7.10	7.76	9.12	**6.16**	**7.52**	**6.96**	**8.32**

The cost is calculated for each year using the portion size identified in the 2007 guide. The costs for adults are highlighted in bold.

**Table 3:** This table provides the average daily cost (in dollars) for each group, as defined in the 2019 Canada Food Guide.

	**Average daily cost using the 2019 Canada food guide in $**							
	**2–3 yrs**	**4–13 yrs**	**Teen (F)**	**Teen (M)**	**Adult (F) 19–50**	**Adult (M) 19–50**	**Adult (F) 50**+	**Adult (M) 50**+
2018	3.20	5.76	6.72	8.00	**6.08**	**7.36**	**6.40**	**7.68**
2019	3.29	5.92	6.91	8.22	**6.25**	**7.56**	**6.58**	**7.89**
2020	3.27	5.88	6.86	8.16	**6.20**	**7.51**	**6.53**	**7.84**
2021	3.51	6.32	7.37	8.78	**6.67**	**8.08**	**7.02**	**8.43**

The cost is calculated for each year using the portion size identified in the 2019 guide. The costs for adults are highlighted in bold.

The main reason for this switch is the merging of dairy and alternatives with meat and alternatives to create the “protein” category. Under the 2007 guide, the adult intake for dairy was between 2 and 3 servings, as was meat. Therefore, on a “total servings” basis, nothing has changed. However, the cost ascribed to each category has changed. Table 4 outlines the costs of milk and meat in the 2007 CFG as well as the cost of proteins in the 2019 CFG. Table 5 provides the minimum, maximum, and mean total costs for both editions of the food guide.

**Table 4:** This table provide a comparison of costs for adults, December 2021, in dollars.

	**Comparison of costs for adults, December 2021, in $**			
	**Adult (F) 19–50**	**Adult (M) 19–50**	**Adult (F) 50**+	**Adult (M) 50**+
**2007**				
Milk, Alt	1.56	1.56	2.34	2.34
Meat, Alt	1.47	2.20	1.47	2.20
Total	3.03	3.76	3.81	4.54
**2019**				
Protein	3.58	4.33	3.76	4.52
Total	3.56	4.33	3.76	4.52

The costs are higher for adults following the 2019 food guide.

**Table 5:** As total cost for each category changes over tim, this table identifies the minimum, the maximum, and the mean total costs in dollars using either the 2007 CFG or the 2019 CFG.

	**Min, Max, and mean for total daily costs (in $)**							
	**2–3 yrs**	**4–13 yrs**	**Teen (F)**	**Teen (M)**	**Adult (F) 19–50**	**Adult (M) 19–50**	**Adult (F) 50**+	**Adult (M) 50**+
**2007**								
Min	3.43	6.43	7.02	8.23	5.54	6.75	6.28	7.49
Max	3.95	7.39	8.08	9.52	6.45	7.89	7.27	8.71
Mean	3.63	6.79	7.43	8.73	5.90	7.20	6.67	7.97
**2019**								
Min	3.16	5.69	6.64	7.90	6.01	7.27	6.32	7.59
Max	3.68	6.62	7.72	9.20	6.99	8.46	7.36	8.83
Mean	3.36	6.05	7.06	8.41	6.39	7.73	6.72	8.07

The costs for adults are bolded in both tables to draw attention to the fact that while it is more affordable for children and teens to follow the 2019 CFG, it is not for adults. There are several reasons for this. The first is that under the 2019 CFG, the amount of protein consumed by adults between the age of 19 and 50 years increases. On a per-portion basis, the average cost of proteins (under both guides, even when split between milk and meat) is the most expensive. To illustrate, in December 2021, the average cost of milk and alternatives was $0.78 per portion; meat and alternatives were $0.73; and protein was $0.75. Even if the average costs for meat and milk are averaged (which works out to be $0.76), there is still a discrepancy of $0.01 because of the actual portion sizes of each food item, which range from 75 g of meat or fish to 250 ml of dairy to 1 egg. While the difference is small, it does add to the costnonetheless.

**Figure 1:**
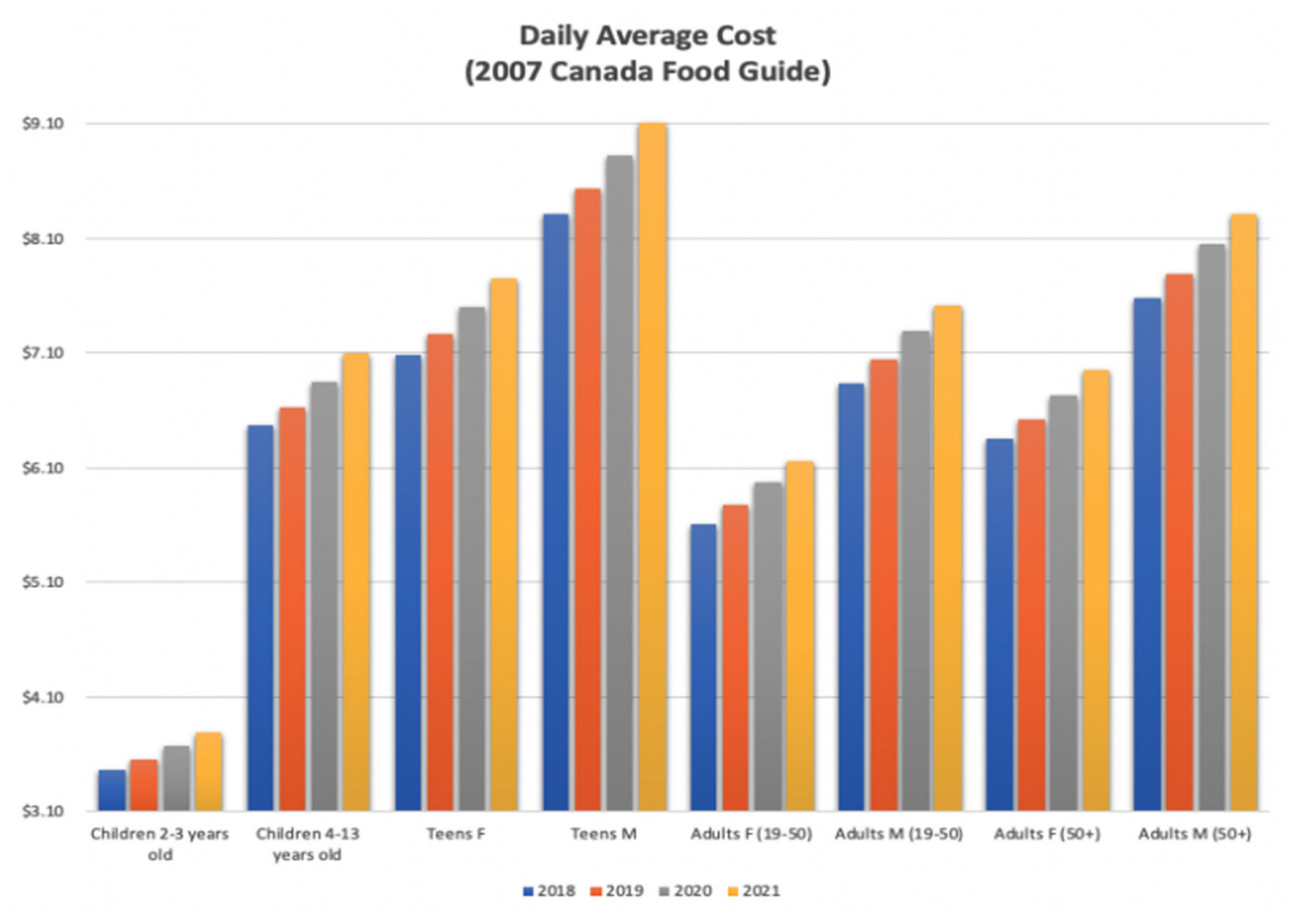
2007 Canada Food Guide daily average cost.

**Figure 2:**
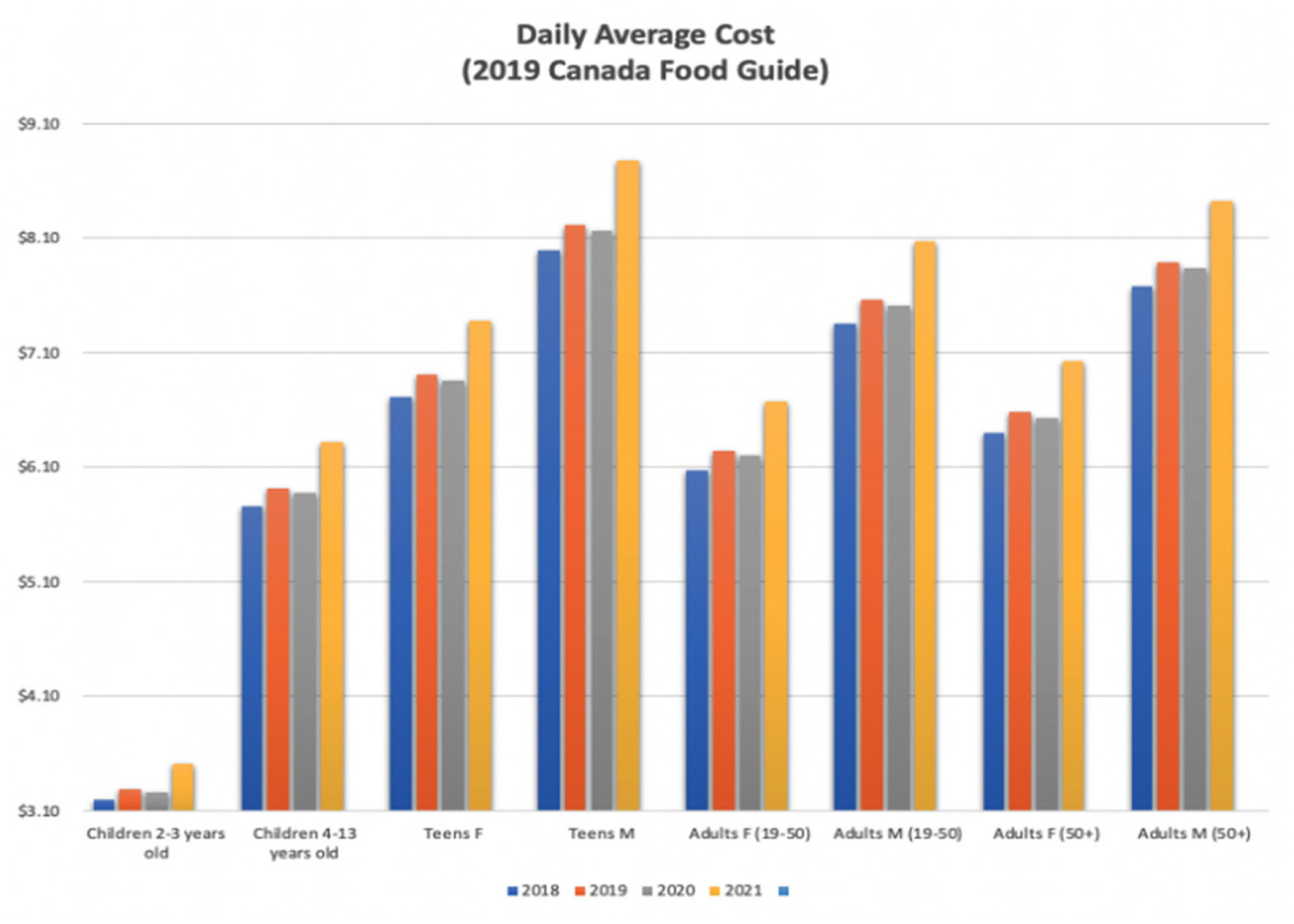
2019 Canada Food Guide daily average cost.

Figures 1, 2 show the daily average cost in graphical format for each age range. Under both food guides, the 2021 costs are the highest for each category. This is quite pronounced when looking at the 2019 CFG results, i.e., the increase in 2021 is larger than that in the 2007 CFG.

It should be noted that for this analysis, we have used the average cost of each food category as there is no “standard food plate” eaten by every individual who uses the food guide. If, for example, a child ate more meat and fish than peanut butter as their source of protein, then following the 2019 guide would be more expensive for that child. Similarly, if an adult ate the reverse (more peanut butter than meat or fish), then it would be less expensive than the average cost of protein, meaning that it would be on par with the 2007 CFG.

## 4. Discussion

We reviewed the gray literature from both the United Nations (UN) and the World Health Organization (WHO) to see if Canada's approach of not considering cost is consistent with that of other countries. According to the Food and Agriculture Association of the United Nations (6), over 100 countries provide food-based guidelines of some kind. Some countries use formal “food guides” while others explicitly state that they do not and instead provide guidance on healthy diets. In reviewing each of the 98 listed country's overview of their guidelines and how they were established, only four countries considered cost: Ethiopia, Italy, Latvia, and Grenada. Ethiopia discussed affordability for both the general population and vulnerable groups (7). Italy indicated that it has considered cost in the selection of the foods included in the guidelines. It went further to consider the “household” level, citing that recommendations were made to help households save money and waste less food (8). Latvia's guidelines “promote seasonal and locally grown produce and low-cost foods” (9). Finally, Grenada cited financial ability as an important limiting factor in access to healthy foods (10).

In 2010, the WHO Global Network of Institutions for Scientific Advice on Nutrition published a meeting report which discusses its approach to the implementation of the recommendations of the WHO Nutrition Program of 2008 (11). The network included the WHO, the National Health and Medical Research Council of Australia, Health Canada, the U.S. Food and Drug Administration, the Food Standards Agency from the United Kingdom, the U.S. Institute of Medicine, Sweden's National Food Administration, the French Food Safety Agency, and the Food Standards for both Australia and New Zealand. Noticeably absent in the report was any consideration of cost or affordability. However, as part of the 60th Session of the Commission for Social Development, the UN and WHO, along with other coalition members, did put forward a *side session* focused on “realizing food and nutrition commitments” during the “Decade of Action on Nutrition,” which runs from 2016 to 2025. As part of that, it does seem that affordability has now become a pillar for moving forward (12), and six policy actions, including the cost-effectiveness of food, were outlined (13).

From the review of the governmental approach, we made an important observation that the consideration of cost and how that cost will affect food security and enable (or rather disable) access to healthy and nutritious food is reactive rather than proactive. Given the events of the past few years, such as COVID-19, the war in Ukraine, supply chain challenges, and climate change, there has been a sharp rise in food inflation around the world, threatening the food security of millions; Canada is no different.

Researchers from around the world are raising important concerns with respect to their government's approach, particularly during the latest struggles with food inflation. We also note that while there is evidence that researchers have and are considering the cost of food in many contexts, ranging from affordability to economic conditions affecting the intake of healthy food (14, 15) to the cost of food as health strategies (16–18), we are focusing on the cost of food in the context of food affordability when eating a diet that adheres to food-based dietary guidelines for this research.

Our analysis demonstrates that in the context of the 2019 CFG, it is *on average* more expensive for adults and less expensive for children and teens to follow than the 2007 CFG. This certainly raises many concerns for users, given that there has been a marked cost increase in other aspects of life in Canada such as gas and housing (19). It is important to remember that the Canada Food Guide is a document that the government specifically creates and disseminates as a guide to healthy eating. Without portion size, the guide is very limited in its capacity to inform in both nutrition and on cost. Given the current economy, users are acutely aware of the cost of food and are struggling, not only in Canada but around the world.

Vandevijvere et al. examined the cost of adhering to Belgium's Food Guide by estimating the daily cost using food-based guidelines in conjunction with the 2014 Growth from Knowledge (GfK) Consumer Can price data (20). Using a sample size of 3,146, they found that while Belgians were adhering to healthy diets more than others, one of their overall conclusions did raise the issue of affordability with a call to improve policies to facilitate greater affordability of healthy diets. Mulik and Haynes-Maslow took a similar approach and used the U.S. Department of Agriculture's food guidelines known as *MyPlate* to estimate the cost of food intake per day for a variety of household makeups including children, teens, single adults, and seniors, as well as the four-person family. In line with the findings of Vandevijvere et al., Mulik and Haynes-Maslow also indicate that the cost of food is not affordable. They determined, for example, that the cost of food for a family of four ranged from $1109 to $1249 per month (21). They also raised a concern that the Supplemental Nutrition Assistance Program (SNAP), which is available to needy families who fall below the gross and net income limits to help pay for food, may not be enough to eat healthily.

Herforth et al. researched the cost of food in Ghana using data collected by the government. Not only did they conclude that their government is not using the data to its full potential to monitor food prices and the effects on the affordability of the Ghanaian diet but also they found that the cost of healthy foods (fruits and vegetables) was more expensive than starchy alternatives and the “cheapest forms of protein foods” (22).

In a Canadian context, there is little research done on the cost of food in relation to the dietary guidelines provided by the CFG. Most of the available research studies focus on the cost of food in relation to something else, such as assessing the food insecurity of a population or subset population in Canada (23, 24) or the cost of food and eating a nutritious diet in the circumpolar regions (25, 26). Researchers also use different definitions of “healthy and nutritious,” and there is an inconsistent basis of information used as not all researchers are using the CFG (27, 28)^4^.

We did, however, find one article by Charlebois et al. that used the CFG as its nutritional basis for determining the cost of the recommended diet (4). Their research, which is pre-pandemic, determined that the 2019 CFG was more affordable than that from 2007. Moreover, in the context of their results, Charlebois et al. also made an important observation: even though the proliferation of the CFG is high in Canada, as it is the second most downloaded document from www.canada.ca, there is little evidence that it is guiding food choices whether they are healthy or not.

The pandemic also affected the way that people oriented themselves to food in many contexts including shopping, food choices, stress eating, meal preparation, and eating with others. In the United States, for example, survey results show that 70% of respondents cooked meals and ate at home rather than out. Diets were healthier and more balanced where “43% of consumers emphasized that they consume more fruits, 42% more vegetables, and 30% more protein-containing foods (meat, chicken, or fish)” (30, 31). A pioneering study on the link between stress and emotional eating was also conducted during the pandemic by Shen et al. which found that there is a statistically significant correlation between perceived stress and emotional eating (32). We term their research as *pioneering* because it is the first to research the linkages between perceived stress, food choices, and emotional eating.

Survey results in France show that their attitudes toward food changed during the lockdown in that they focused on necessities, food preparation, and cost, which ultimately had a positive impact on food waste (33). In Poland, a study demonstrated that high school students' attitudes toward healthy food were positively affected during the pandemic with a new focus on a healthy diet. This “may have increased the importance of health and weight control” for teens (34).

In Canada, there was an increase in home food gardening during the pandemic (35, 36). Studies also show that the pandemic may have increased food insecurity, particularly for children, those who stopped working during the pandemic (either by choice or by necessity), and those with job instability (37). A study by Labonte and Nielsen found that during the pandemic, families “placed great importance on food convenience” due to time constraints and unavoidable restrictions such as children attending school online while parents also worked at home (38). Conversely, those living alone used cooking and meal preparation as a “coping strategy” (38).

It is clear that the pandemic has changed people's attitudes toward food and raised concerns regarding affordability—a message which does not seem to impact or move leaders into action. While there is evidence that some countries have considered the cost of a healthy diet, the changes in the food system brought on by the pandemic have presented Canada with an opportunity to be a world leader in providing the country with nutritional guidance that is both based on science and the economy. Consideration of both portion sizing and the cost of nutritional food needs to be at the forefront of Canada's next food guide to meet the needs of Canadians and foster a healthy nation.

## 5. Conclusion and future work

While the 2019 CFG may have been more affordable in the pre-pandemic era for all age categories, our research shows that this is no longer the case. The merging of dairy and meat into proteins was favorable, from a cost perspective, for children and teens. This merging was not favorable for adults, as it only increased the costs. Since the pandemic, access to proteins has become more expensive. Moreover, since the 2019 CFG was released, Canada has and continues to experience high food inflation, which has further worsened the affordability of the 2019 CFG.

Similar to Charlebois et al., we also concluded that cost was not sufficiently considered in the creation of the 2019 CFG, raising significant concerns, not only for those who use the CFG on an individual/familial level to cost out foods that will conform with the guidelines, but also for institutions who rely on the guidance in the CFG to create budgets.

Food inflation continues to rise even though usual contributors to food (and overall inflation) such as gas prices have fallen. This has also raised concerns in Canada about “greedflation,” a term often used to describe unjustified increases in food retail prices. While there is no evidence of “greedflation” based on the publicly available data (39), the narrative and concerns continue.

There are several significant gaps that need to be filled in future works. Although some works have been done on adherence to the CFG (40, 41), these studies have gone beyond the CFG and also looked into a specific subset of the population. To fully understand what adherence truly is, a Canada-wide study needs to be done to determine to what extent and what capacity Canadians have to follow the food guide. Both nutrition and the cost of that nutrition for the different peoples of Canada need to be considered.

We would like to extend our current study to look at the individual provinces as it is important to determine which areas of Canada are most greatly affected by food inflation. Statistics Canada does not make any data available for food in the territories. This is a significant gap in the data and severely limits the investigation and analysis that can be done on the North. Therefore, we would also like to develop two studies that will focus on the territories as well as the circumpolar region to determine the effects of food inflation there.

We would also like to analyze if there are any concerns from corporate entities with regard to food donation lawsuits. As part of this, we aim to determine how much food is being donated by grocers, markets, and other food-growing operations. We also plan to examine how best before dates and expiry dates are affecting donations as well as how these types of regulations are affecting the overall price of food.

Finally, we would like to examine how food grading is affecting the cost of food and how the food that “doesn't make the grade”—which is still perfectly edible and safe to eat—is being handled (i.e., if it is being donated or left to spoil).

## Data availability statement

The raw data supporting the conclusions of this article will be made available by the authors, without undue reservation.

## Author contributions

ST led the study conception and design, gathered the data and analyzed it, and drafted the manuscript. SC was also involved in the study conception, reviewed the data analysis, and reviewed the manuscript. ST and SC approved the final manuscript. JM oversaw the project administration. All authors contributed to the article and approved the submitted version.

## Footnotes

^1^The only response to concerns regarding affordability and cost that we could find was the following: “...a range of nutritious foods form the foundation of healthy eating: frozen, packaged and canned products are convenient and nutritious options, especially when fresh food is out of season, more costly or unavailable.” This does not actually address the affordability or cost concerns raised during the consultation phase as the cost for frozen, packaged, and/or canned products has not been reasonably considered as there is no evaluation of what that costs is in relation to out-of-season, more costly, or unavailable food. https://www.canada.ca/en/services/health/publications/food-nutrition/canada-food-guide-phase2-what-we-heard.html.

^2^ https://www150.statcan.gc.ca/t1/tbl1/en/tv.action?pid=1810000201

^3^The most current data released by Statistics Canada only goes to February 2022 at the time of this research.

^4^It should be noted that often in studies, Canada's Nutritious Food Basket has been used as the basis for determining healthy foods. In 2019, however, the Nutritious Food Basket was updated to be in-line with the Canada Food Guide, which alleviates the disconnect between the food basket and the food guide (29).

## References

[1] Canada H. Canada's Food Guide. Available online at: https://www.canada.ca/en/health-canada/services/canada-food-guide/about/history-food-guide.html (accessed October 28, 2022).

[2] Canada H. Canada's Food Guide. (2019).

[3] Health Link BC. Canada's Food Guide FAQs. Available online at: https://www.healthlinkbc.ca/healthy-eating-physical-activity/food-and-nutrition/canadas-food-guide-faqs#:~:text=Policymakers%2C%20health%20professionals%2C%20and%20institutions,to%20teach%20about%20healthy%20eating (accessed October 28, 2022).

[4] Charlebois S Smook M Wambui BN Somogyi S Racey M Fiander D. Can Canadians afford the new Canada' s food guide? Assessing barriers and challenges. J Food Res. (2021) 10:1–22. 10.5539/jfr.v10n6p22

[5] Canada S. Monthly average retail prices for food and other selected products. Available online at: https://www150.statcan.gc.ca/t1/tbl1/en/tv.action?pid=1810000201 (accessed October 28, 2022).

[6] Food Nations Nations AA of the U. Food-based dietary guidelines. Available online at: https://www.fao.org/nutrition/nutrition-education/food-dietary-guidelines/en/ (accessed October 28, 2022).

[7] Institute EPH. Food-based dietary guidelines - Ethiopia. Available online at: https://www.fao.org/nutrition/education/food-dietary-guidelines/regions/countries/ethiopia/en/ (accessed October 28, 2022).

[8] Food C Centre NR. Food-based dietary guidelines - Italy. Available online at: https://www.fao.org/nutrition/education/food-dietary-guidelines/regions/countries/italy/en/ (accessed October 28, 2022).

[9] Health M of. Food-based dietary guidelines - Latvia. Available online at: https://www.fao.org/nutrition/education/food-dietary-guidelines/regions/countries/latvia/en/ (accessed October 28, 2022).

[10] Food G Council N. Food-based dietary guidelines - Grenada. Available online at: https://www.fao.org/nutrition/education/food-dietary-guidelines/regions/countries/grenada/en/ (accessed October 28, 2022).

[11] Organization WH. WHO *Global Network of Institutions for Scientific Advice on Nutrition: Report of the First Meeting, 11-12 March 2010*. Geneva, Switzerland:WHO (2010).

[12] Organization WH. Working together in 2022 towards realizing food and nutrition commitments. Available online at: https://www.who.int/news-room/events/detail/2022/02/09/default-calendar/working-together-in-2022-towards-realizing-food-and-nutrition-commitments (accessed October 28, 2022).

[13] Branca F. Nutrition Outcomes of the UN Food Systems Summit. Available online at: https://cdn.who.int/media/docs/default-source/nutritionlibrary/events/2022/nutrition-outcomes-of-the-unfss-presentation.pdf?sfvrsn=df4ec574_7 (accessed October 28, 2022).

[14] Milicic S DeCicca P. The impact of economic conditions on healthy dietary intake: evidence from fluctuations in Canadian unemployment rates. J Nutri Edu Behav. (2017) 49:632–8. 10.1016/j.jneb.2017.06.010 28889852

[15] Musaiger AO. Socio-cultural and economic factors affecting food consumption patterns in the Arab countries. J R Soc Health. (1993) 113:68–74. 10.1177/146642409311300205 8478894

[16] Brunner E Cohen D Toon L. Cost effectiveness of cardiovascular disease prevention strategies: a perspective on EU food based dietary guidelines. Public Health Nutri. (2001) 4:711–5. 10.1079/PHN2001161 11683566

[17] Pineda E Brunner EJ Llewellyn CH Mindell JS. The retail food environment and its association with body mass index in Mexico. Int J Obes. (2021) 45:1215–28. 10.1038/s41366-021-00760-2 33597735 PMC8159738

[18] Van der Horst K Brunner TA Siegrist M. Ready-meal consumption: associations with weight status and cooking skills. Public Health Nutr. (2011) 14:239–45. 10.1017/S1368980010002624 20923598

[19] Statistics Canada. Rising prices are affecting the ability to meet day-to-day expenses for most Canadians. Available online at: https://www150.statcan.gc.ca/n1/daily-quotidien/220609/dq220609a-eng.htm (accessed October 28, 2022).

[20] Vandevijvere S De Ridder K Drieskens S Charafeddine R Berete F Demarest S. Food insecurity and its association with changes in nutritional habits among adults during the COVID-19 confinement measures in Belgium. Public Health Nutr. (2021) 24:950–6. 10.1017/S1368980020005005 33292888 PMC7804079

[21] Mulik K Haynes-Maslow L. The affordability of MyPlate: An analysis of SNAP benefits and the actual cost of eating according to the dietary guidelines. J *Nutri Edu Behav*. (2017) 49:623–31. 10.1016/j.jneb.2017.06.005 28889851

[22] Herforth A Masters W Bai Y Sarpong D. The cost of recommended diets: Development and application a food Price index based on food-based dietary guidelines (P10-033-19). Curr Develop Nutri. (2019) 3(Supplement_1):nzz034-P10. 10.1093/cdn/nzz034.P10-033-1929523173

[23] Larsen K Gilliland J. A farmers' market in a food desert: evaluating impacts on the price and availability of healthy food. Health Place. (2009) 15:1158–62. 10.1016/j.healthplace.2009.06.007 19631571

[24] Socha T Chambers L Zahaf M Abraham R Fiddler T. Food availability, food store management, and food pricing in a northern community first nation community. Int J Human Soc Sci. (2011) 1:49–61.

[25] Galloway T. Canada's northern food subsidy nutrition North Canada: a comprehensive program evaluation. Int J Circumpolar Health. (2017) 76:1279451. 10.1080/22423982.2017.1279451 28151097 PMC5328347

[26] Kenny TA Fillion M MacLean J Wesche SD Chan HM. Calories are cheap, nutrients are expensive–the challenge of healthy living in arctic communities. Food Policy. (2018) 80:39–54. 10.1016/j.foodpol.2018.08.006

[27] MacDonald BJ Andrews D Brown RL. The Canadian Elder Standard–Pricing the cost of basic needs for the Canadian elderly. Can J Aging/La Revue canadienne du vieillissement. (2010) 29:39–56. 10.1017/S0714980809990432 20202264

[28] Herrick KA Ogden CL. Nutrition surveillance. In: Present Knowledge in Nutrition. Elsevier (2020). p. 217–33. 10.1016/B978-0-12-818460-8.00012-5

[29] Canada H. National Nutritious Food Basket. Available online at: https://www.canada.ca/en/health-canada/services/food-nutrition/food-nutrition-surveillance/national-nutritious-food-basket.html (accessed October 28, 2022).

[30] Aday S Aday MS. Impact of COVID-19 on the food supply chain. Food Quality and Safety. (2020) 4:167–80. 10.1093/fqsafe/fyaa024 PMC7499675 40504224

[31] DeBroff S. Has Impacted Consumer Food Habits (2020).

[32] Shen W Long LM Shih CH Ludy MJ. A humanities-based explanation for the effects of emotional eating and perceived stress on food choice motives during the COVID-19 pandemic. Nutrients. (2020) 12:2712. 10.3390/nu12092712 32899861 PMC7551550

[33] Askew K. Life in lockdown: Coronavirus prompts half of French consumers to reappraise “value” of food [Online]. Available online at: https://www.foodnavigator.com/Article/2020/05/29/Life-in-lockdown-Coronavirus-prompts-half-of-French-consumers-to-reappraise-value-of-food (accessed May 29, 2020).

[34] Glabska D Skolmowska D Guzek D. Population-based study of the changes in the food choice determinants of secondary school students: Polish adolescents' COVID-19 experience (place-19) study. Nutrients. (2020) 12:2640. 10.3390/nu12092640 32872577 PMC7551462

[35] Mullins L Charlebois S Finch E Music J. Home food gardening in Canada in response to the COVID-19 pandemic. Sustainability. (2021) 13:3056. 10.3390/su1306305635990764

[36] Music J Mullins L Charlebois S Large C Mayhew K. Seeds and the city: a review of municipal home food gardening programs in Canada in response to the COVID-19 pandemic. Human Soc Sci Commun. (2022) 9:1–12. 10.1057/s41599-022-01301-6 35990764 PMC9381390

[37] Idzerda L Gariépy G Corrin T Tarasuk V McIntyre L Neil-Sztramko S. What is known about the prevalence of household food insecurity in Canada during the COVID-19 pandemic: a systematic review. Health Promotion and Chronic Disease Prevention in Canada: *Res Pol Pract*. (2022) 42:1. 10.24095/hpcdp.42.5.01 35420755 PMC9306322

[38] Labonte K Nielsen DE. Food purchasing, food values, and food skills over the course of the covid-19 pandemic: evaluation of patterns according to household structure in Quebec, Canada. J Home Econ Inst Aust. (2022) 27:27–41.

[39] Charlebois S Taylor S Taylor S Music J. Dealing with “greedflation” - Part II. Dalhousie Agri-Food Analytics Lab (2022).

[40] Brassard D Elvidge Munene LA St-Pierre S Guenther PM Kirkpatrick SI Slater J. Development of the healthy eating food index (HEFI)-2019 measuring adherence to Canada's food guide 2019 recommendations on healthy food choices. Appl Physiol Nutr Metab. (2022) 47:595–610. 10.1139/apnm-2021-0415 35030038

[41] Allen JP Taylor JG Rozwadowski MM Boyko JA Blackburn DF. Adherence to Canada's food guide among pharmacy students. Can Pharm J. (2011) 144:79–84. 10.3821/1913-701X-144.2.79

